# Comparative Analysis of the Discriminatory Performance of Different Well-Known Risk Assessment Scores for Extended Hepatectomy

**DOI:** 10.1038/s41598-020-57748-7

**Published:** 2020-01-22

**Authors:** Omid Ghamarnejad, Elias Khajeh, Nahid Rezaei, Khashayar Afshari, Ali Adelian, Mohammadsadegh Nikdad, Katrin Hoffmann, Arianeb Mehrabi

**Affiliations:** Department of General, Visceral, and Transplantation Surgery, University of Heidelberg, Heidelberg, 69120 Germany

**Keywords:** Surgical oncology, Risk factors

## Abstract

The aim of this study was to assess and compare the discriminatory performance of well-known risk assessment scores in predicting mortality risk after extended hepatectomy (EH). A series of 250 patients who underwent EH (≥5 segments resection) were evaluated. Aspartate aminotransferase-to-platelet ratio index (APRI), albumin to bilirubin (ALBI) grade, predictive score developed by Breitenstein *et al*., liver fibrosis (FIB-4) index, and Heidelberg reference lines charting were used to compute cut-off values, and the sensitivity and specificity of each risk assessment score for predicting mortality were also calculated. Major morbidity and 90-day mortality after EH increased with increasing risk scores. APRI (86%), ALBI (86%), Heidelberg score (81%), and FIB-4 index (79%) had the highest sensitivity for 90-day mortality. However, only the FIB-4 index and Heidelberg score had an acceptable specificity (70% and 65%, respectively). A two-stage risk assessment strategy (Heidelberg–FIB-4 model) with a sensitivity of 70% and a specificity 86% for 90-day mortality was proposed. There is no single specific risk assessment score for patients who undergo EH. A two-stage screening strategy using Heidelberg score and FIB-4 index was proposed to predict mortality after major liver resection.

## Introduction

Extended hepatectomy (EH) is the only potentially curative treatment for bilobar or large liver lesions^1,2^. Recent developments in the field of hepatobiliary surgery have increased the indications for EH and it has become a standard surgical procedure in most high-volume centers. Despite improvements, the rates of morbidity and mortality after EH remain high^3,4^. A comprehensive preoperative assessment and proper patient selection criteria may predict and reduce the risk of postoperative morbidity and mortality. This would improve patient selection and patient management, and thereby improve the intraoperative findings and postoperative outcomes of EH.

Several risk assessment scores have been proposed to predict outcomes after liver resection^3,5–9^. However, most are based on postoperative parameters. Because the phase shortly after EH is critical, many patients may not benefit from a risk assessment that is based on postoperative data. Some preoperative risk scoring systems have been introduced^10–13^, but their discriminatory performance have not been evaluated and compared exclusively in patients undergoing EH, who have a relatively higher risk of postoperative morbidity and mortality than those undergoing minor hepatectomy.

The aim of this study was to evaluate the ability of well-known risk assessment scores to predict mortality risk after EH. All included risk assessments have been developed or validated in large cohorts of liver resection patients and have been published in high-impact hepatobiliary surgery or hepatogastroenterology journals. A second aim was to propose a risk assessment strategy for patients undergoing EH based on these risk scores.

## Results

### Patient collective

The demographic and baseline data were compared, as well as perioperative outcomes between patient risk assessment scores that were calculated with incomplete data (n = 26, 9.4%) and complete data. Baseline data and outcomes did not differ significantly between the two groups (data not shown). After excluding patients with incomplete data, 250 patients were included in the final analysis. The mean age of included patients was 60 ± 12 years (range: 18–86 years old) and 134 patients (53.6%) were male. The most common indication for EH was primary liver malignancy in 136 patients (54.4%), followed by liver metastasis in 80 patients (32.0%), and benign indications in 34 patients (13.6%). One hundred and four patients (43.3%) received preoperative systemic chemotherapy. Baseline clinical and demographic characteristics, as well as preoperative laboratory data, are shown in Tables 1 and 2.

**Table 1:** Demographic and preoperative clinical data.

Variables	Total (n = 250) n (%) or mean ± SD
Age, years	60 ± 12
Sex	
(male/female)	134/116
BMI (kg/m^2^)	25.4 ± 4.4
Diabetes mellitus	24 (9.6)
ASA score	
Class 1	10 (4.0)
Class 2	130 (52.0)
Class 3	110 (44.0)
Indication of hepatectomy	
Benign liver disease	34 (13.6)
Primary malignancy	136 (54.4)
Cholangiocarcinoma	115 (46.0)
Hepatocellular carcinoma	21 (8.4)
Metastatic disease	80 (32.0)
Preoperative chemotherapy	104 (43.3)

BMI: body mass index; ASA: American Society of Anesthesiologists; SD: standard deviation.

**Table 2:** Preoperative laboratory data.

Variables	Total (n = 250) mean ± standard deviation
Sodium (mmol/l)	138.6 ± 2.9
Creatinine (mg/dl)	0.8 ± 0.2
Aspartate aminotransferase (U/l)	74.1 ± 93.9
Alanine aminotransferase (U/l)	87.5 ± 120.1
Gamma-glutamyl transferase (U/l)	329.9 ± 484.9
International normalized ratio	1.0 ± 1.6
Total bilirubin (mg/dl)	2.1 ± 3.3
Albumin (g/l)	39.3 ± 7.9
Platelet (n/l)	296.1 ± 130.4

### Discriminatory value of predictive risk scores

**Figure 1:**
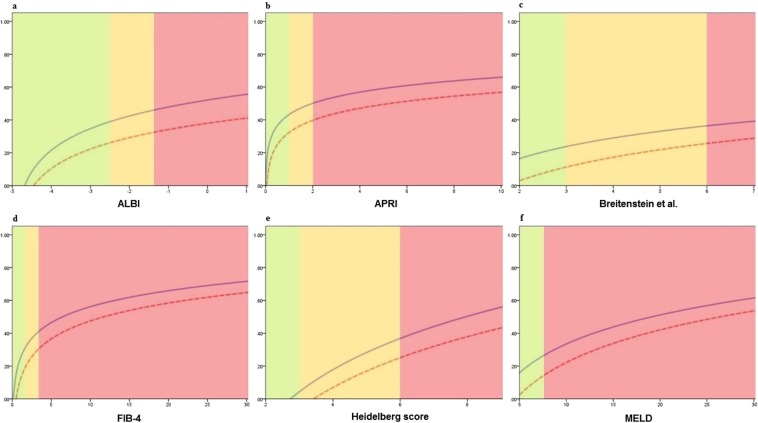
The incidence of major morbidity (–) and 90-day mortality (–) in low- (green), intermediate- (yellow), and high-risk (red) patients based on different risk assessment scores. APRI, aminotransferase-to-platelet ratio index; ALBI, albumin to bilirubin grade; FIB-4, liver fibrosis index; MELD, model for end-stage liver disease.

Curve estimation was applied to show changes in the proportion of major morbidity and 90-day mortality with increasing risk. The low-, intermediate-, and high-risk groups are shown in green, yellow, and red respectively in Fig. 1 according to the predefined cut-off points of each risk assessment score (Table 3). As expected, the major morbidity and 90-day mortality after EH increased with increasing value in all risk scores. As shown in Table 4, except for ALBI, the rate of major morbidity and 90-day mortality significantly differed between low-, intermediate-, and high-risk patients based on the proposed cut-off values of all risk scores. The FIB-4 index showed the highest increase in major morbidity and 90-day mortality (40% for both) from the low-risk group to the high-risk group.

**Table 3:** Details of the selected risk assessment scores.

Score	Description	Formula	Risk categories	Cut-offs
ALBI grade	The ALBI was introduced to assess liver function in patients with hepatocellular carcinoma^39^. It was also shown that the ALBI grade can assess the risk of post-hepatectomy mortality in hepatocellular carcinoma patients^13^.	(Log_10_ total bilirubin × 0.66) − (albumin × 0.085)	Low	≤−2.6
			Intermediate	−2.59 to −1.39
			High	>−1.39
APRI	The APRI was first introduced as a predictive measure for fibrosis and cirrhosis in patients with chronic hepatitis^25^. Different studies also demonstrated that APRI can significantly predict post-hepatectomy morbidity and mortality^26–28^	(AST/the upper limit of normal value) × 100 ÷ platelet (10^9^/L)	Low	≤1
			Intermediate	1 to 2
			High	≥2
Breitenstein score	This score was introduced by Breitenstein *et al*.^31^ to predict post-hepatectomy poor outcomes in non-cirrhotic patients.	One point is assigned for ASA III and IV, three points are assigned for AST ≥ 40 U/L, two points are assigned for major (extensive) liver resection, four points are assigned for extrahepatic procedures	Low	<3
			Intermediate	3 to 5
			High	≥6
FIB-4 index	The FIB-4 index is considered a valid measure for assessing liver fibrosis in different liver diseases^30^. However, it was shown that this index could also be used to predict post-hepatectomy mortality^12^.	Age × AST/platelet count [×10^3^/µL] × ALT^1/2^	Low	≤1.45
			Intermediate	1.46 to 3.25
			High	>3.25
Heidelberg score	Was introduced as a prognostic risk score for post-hepatectomy morbidity and mortality, and was also externally validated in a cohort of 281 patients in another center^29^.	One point is assigned for age ≥ 60 years, right trisectionectomy, preoperative INR ≥ 1.1, preoperative GGT ≥ 60 U/L, intrahepatic cholangiocarcinoma, and ASA III. Two points are assigned for preoperative platelet count ≤ 120/nL, and perihilar cholangiocarcinoma. Three points are assigned for preoperative creatinine value ≥ 2 mg/dL. Five points are assigned for ASA IV	Low	≤3
			Intermediate	4 to 5
			High	≥6
MELD score	The MELD score was originally introduced as an assessment measure for the intensity of chronic liver conditions^32^. The prognostic value of MELD score was also validated for post-hepatectomy morbidity and mortality in patients with primary^34^ and secondary^33^ liver malignancies.	3.78 × ln[serum bilirubin (mg/dL)] + 11.2 × ln[INR] + 9.57 × ln[serum creatinine (mg/dL)] + 6.43	Low	≤7.24
			High	>7.24

APRI, aminotransferase-to-platelet ratio index; ALBI, albumin to bilirubin grade; FIB-4, liver fibrosis index; MELD, model for end-stage liver disease; AST, aspartate aminotransferase; ASA: American Society of Anesthesiologists; INR, international normalized ratio; GGT, gamma-glutamyl transferase.

**Table 4:** The rate of morbidity and mortality in the risk categories of risk assessment scores.

Scores	Risk categories	Total (n = 250) n (%)	Major morbidity n (%)	*p* value	90-day mortality n (%)	*p* value
ALBI grade	Low	205 (82.0)	54 (26.3)	0.158	30 (14.6)	0.069
	Intermediate	39 (15.6)	15 (38.5)		11 (28.2)	
	High	6 (2.4)	3 (50.0)		2 (33.3)	
APRI	Low	54 (21.6)	9 (16.7)	**0.002**	3 (5.6)	**<0.001**
	Intermediate	66 (26.4)	13 (19.7)		5 (7.6)	
	High	130 (52.0)	50 (38.5)		35 (26.9)	
Breitenstein score	Low	47 (18.8)	7 (14.9)	**0.036**	2 (4.3)	**<0.001**
	Intermediate	111 (44.4)	32 (28.8)		14 (12.6)	
	High	92 (36.8)	33 (35.9)		27 (29.3)	
FIB-4 index	Low	141 (56.4)	25 (17.7)	**<0.001**	8 (5.7)	**<0.001**
	Intermediate	67 (26.8)	22 (32.8)		16 (23.9)	
	High	42 (16.8)	25 (59.5)		19 (45.2)	
Heidelberg score	Low	32 (12.8)	2 (6.3)	**<0.001**	0 (0.0)	**<0.001**
	Intermediate	111 (44.4)	22 (19.8)		8 (7.2)	
	High	107 (42.8)	48 (44.9)		35 (32.7)	
MELD score*	Low	123 (49.2)	26 (21.1)	**0.012**	11 (8.9)	**0.001**
	High	127 (50.8)	46 (36.2)		32 (25.2)	

APRI, aminotransferase-to-platelet ratio index; ALBI, albumin to bilirubin grade; FIB-4, liver fibrosis index; MELD, model for end-stage liver disease.

*No intermediate-risk group is defined in the MELD score.

### Prediction of 90-day mortality

**Figure 2:**
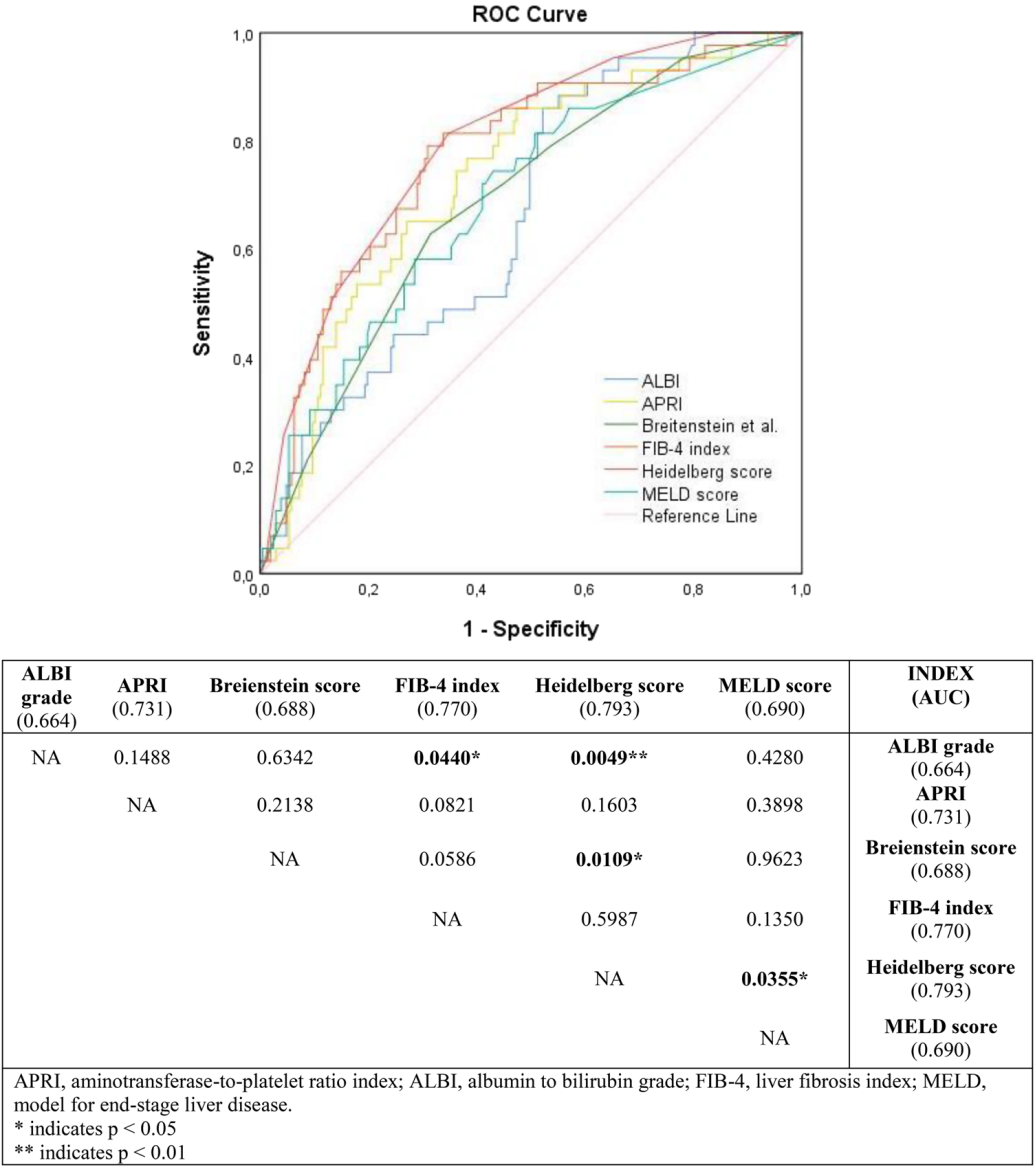
Area under the curve (AUC) for each risk assessment score for discrimination of 90-day mortality. APRI, aminotransferase-to-platelet ratio index; ALBI, albumin to bilirubin grade; FIB-4, liver fibrosis index; MELD, model for end-stage liver disease.

**Figure 3:**
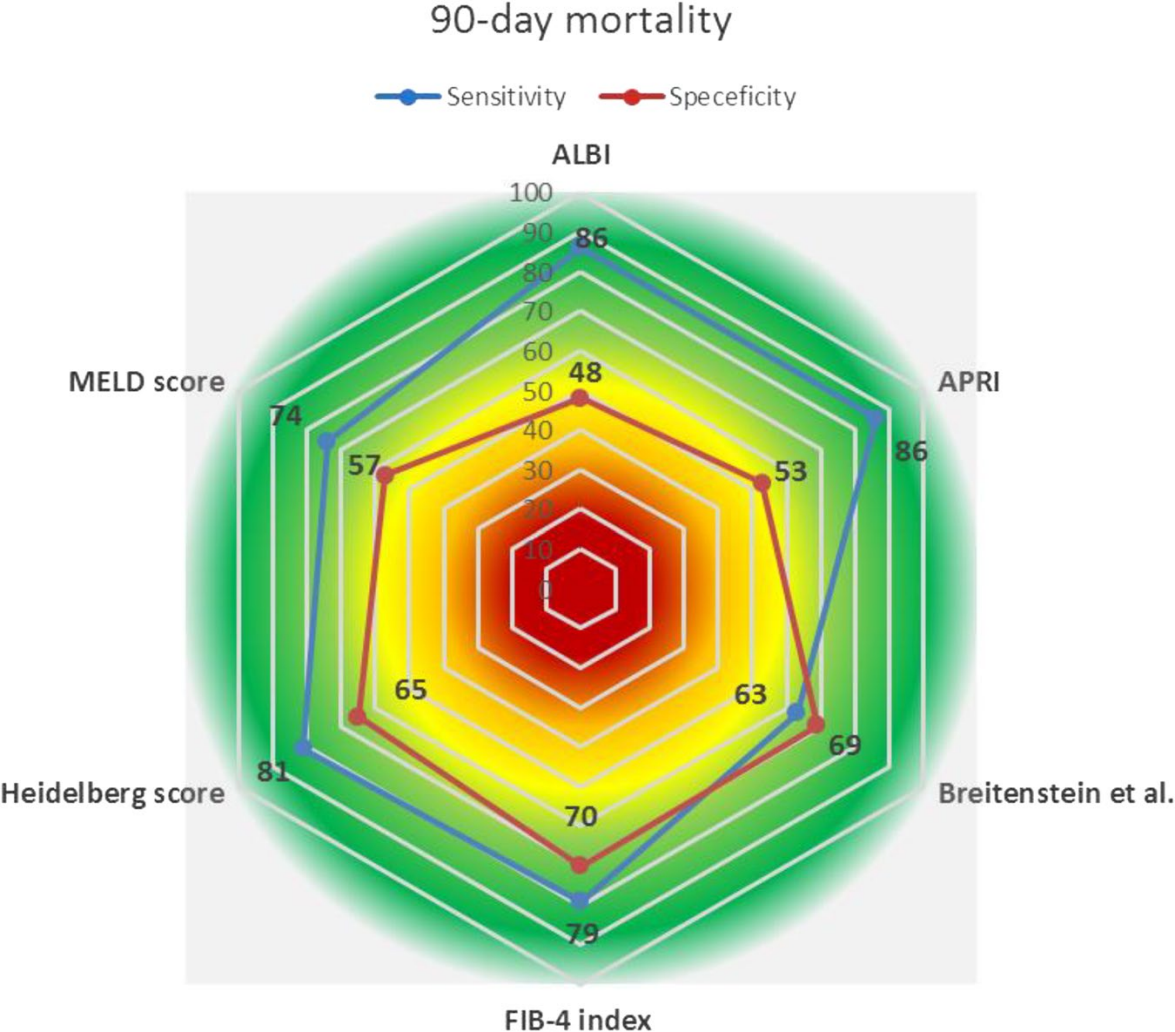
Sensitivity (blue) and specificity (orange) of each risk assessment score for predicting 90-day mortality. APRI, aminotransferase-to-platelet ratio index; ALBI, albumin to bilirubin grade; FIB-4, liver fibrosis index; MELD, model for end-stage liver disease.

Based on the receiver operating characteristic (ROC) curve analysis (Fig. 2), the Heidelberg score (area under the curve [AUC] = 79%), FIB-4 index (AUC = 77%), and APRI (AUC = 73%) had AUCs more than 70%, and the model for end-stage liver disease (MELD) score (AUC = 69%), Breitenstein score (AUC = 69%), and ALBI score (AUC = 66%) had AUCs between 60% and 70% (all *p* < 0.01). The estimated cut-off values for ALBI, APRI, Breitenstein score, FIB-4 index, Heidelberg score, and MELD score were −3.74, 0.19, 5.50, 1.52, 5.50, and 7.38, respectively. Figure 3 shows the sensitivity and specificity of risk scores for 90-day mortality based on the estimated cut-offs. APRI (86%), ALBI grade (86%), Heidelberg score (81%), and FIB-4 index (79%) had the highest sensitivity for 90-day mortality. However, only the FIB-4 index and Heidelberg score had an acceptable specificity of 70% and 65%, respectively. The APRI showed a specificity of 53% and the ALBI grade a specificity of 48% for 90-day mortality.

To compare the discriminatory ability of different risk scores for 90-day mortality, pairwise comparison of AUCs was performed. As shown in Fig. 2, the Heidelberg risk score performed better than the ALBI grade, Breitenstein score, and MELD score but there were no significant differences between the discriminatory abilities of the Heidelberg score compared with the APRI and FIB-4 index. There was also no significant difference in discriminatory ability between other risk scores. Accordingly, the Heidelberg score, APRI, and FIB-4 index were selected to propose a risk assessment strategy for patients undergoing EH.

### Proposed risk assessment strategy for EH

Based on the determined sensitivity and specificity of the selected risk scores, a two-stage screening method was proposed. A high sensitivity test (Heidelberg and APRI scores) was selected for the first stage and a high specificity test (FIB-4 index) was selected for the second stage. Two risk assessment strategies were assessed:

1. A two-stage risk assessment strategy using the Heidelberg score as the first and the FIB-4 index as the second screening test.
2. A two-stage risk assessment strategy using the APRI score as the first and the FIB-4 index as the second screening test.

#### Heidelberg−FIB-4 model

In this proposed model, all patients were first screened with the Heidelberg score, and those whose risk score was < 5.50 were considered low-risk. High-risk patients (risk score ≥ 5.50) underwent a second, more specific screening with the FIB-4 index, and those whose FIB-4 index was < 1.52 were considered acceptable risk. Patients who were considered high risk by both tests were assumed to be at high risk of mortality after surgery. The overall sensitivity and specificity of this two-stage risk assessment strategy are 70% and 86%, respectively.

#### APRI—FIB-4 model

We also tested the combination of APRI (first screening) and FIB-4 index (second screening) using the same method. This stepwise risk assessment strategy has an overall sensitivity of 77% and an overall specificity of 72%.

## Discussion

Extended liver resection is the only curative treatment for patients with multiple or large tumors that can prolong survival^14–20^. However, inadequate liver remnant due to EH can cause serious complications such as post-hepatectomy liver failure and poses a significant risk of morbidity and mortality^21–23^. Therefore, proper patient selection is crucial to improve post-EH outcomes^24^. Considering the importance of patient selection, different preoperative risk assessment scores have been proposed to improve the efficacy of the operation and prognosis. However, these criteria have not been comprehensively compared and their sensitivity and specificity have not been simultaneously evaluated in a homogenous population of patients undergoing EH. Hence, in the current study, we compared well-known risk assessment scores and evaluated their ability to predict mortality in patients undergoing EH.

The results of the present study indicate that, in the absence of EH-specific risk assessment scores, some existing risk assessment methods can predict the outcomes after EH with acceptable discriminatory ability. However, the sensitivity and specificity of these scores were heterogenic and there was no agreement in the selection of high-risk patients. The APRI, ALBI grade, Heidelberg score, and FIB-4 index were the most sensitive, and the FIB-4 index was the most specific predictive score for mortality after EH.

The APRI was introduced by Wai *et al*. in 2003 as a predictive measure for fibrosis and cirrhosis in patients with chronic hepatitis^25^. Later, different studies concluded that its preoperative measures significantly predict post-hepatectomy morbidity and mortality^26–28^. Mai *et al*. recently evaluated 1,044 hepatocellular carcinoma patients that underwent liver resection, and demonstrated that APRI could significantly predict post-hepatectomy outcomes (AUC = 0.743), in agreement with our results. This confirms the ability of the APRI to predict mortality after EH^27^.

Hoffman *et al*. evaluated patient- and procedure-related factors that affect postoperative morbidity and mortality after liver resection. They reviewed the records of 1,796 patients that underwent liver resection, and showed that age, extension of planned liver resection, preoperative platelet count, international normalized ratio (INR), g-GT, creatinine levels, histologic tumor diagnosis, and ASA classification were significantly associated with post-surgical morbidity and mortality. They introduced the Heidelberg score as a prognostic risk score, which was externally validated in 281 patients and had an AUC of 0.866^29^. Results of the current study showed a similar ability of the Heidelberg score (AUC of 0.793) to predict mortality in patients undergoing EH.

The FIB-4 index is considered a valid measure for assessing liver fibrosis in different liver diseases^12,30^. Toyoda *et al*. indicated that this index could also be used to predict long-term post-curative hepatectomy outcomes; they showed that higher FIB-4 measures were associated with a significant increase in mortality^12^. In the current study, we show that this measure can significantly predict 90-day mortality after EH.

Wang *et al*. have demonstrated that the ALBI grade can assess the risk of post-hepatectomy mortality in hepatocellular carcinoma patients (AUC = 0.607)^13^. However, our results suggest that the ALBI is better able to predict mortality after EH (AUC = 0.664).

Breitenstein *et al*.^31^ studied 615 hepatectomy cases in a single center. They introduced a calibrated scoring index to predict poor post-hepatectomy outcomes in non-cirrhotic patients that was based on the odds ratios for preoperative AST levels, ASA grade, extension of resection, and extrahepatic procedures during surgery. Their scoring system, including three different risk levels, enhanced the accuracy of decision making by considering individual patient characteristics, and indicated the costs of health care. In the current study, we have shown that the Breitenstein score can predict 90-day mortality after EH, with a sensitivity and specificity above 65%.

The MELD score was originally introduced as an assessment measure for the intensity of chronic liver conditions, but can also calculate the risks of hepatectomy in patients with liver metastasis^32^. Mortality risk was more than two times higher in patients with a MELD score > 7.24^33^. This index has also been validated for post-hepatectomy complications in cirrhotic patients with hepatocellular carcinoma (AUC = 0.85)^33,34^. Data from the present study supports the significant accuracy of the MELD score in predicting mortality after EH (AUC = 0.690).

**Figure 4:**
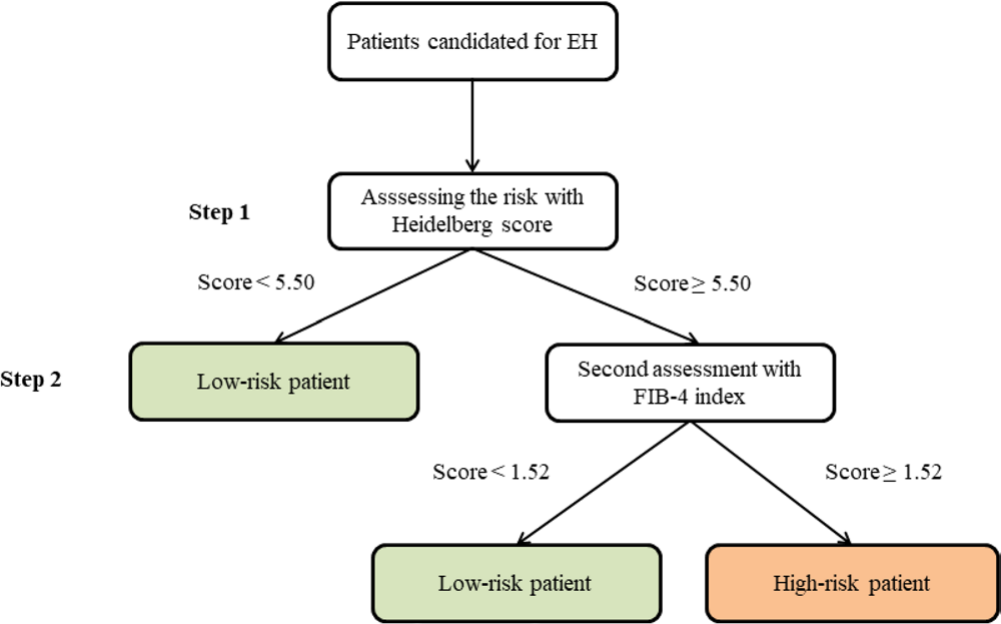
A proposed risk assessment strategy for patients undergoing major liver resection.

The risk scores investigated in this study have been validated in large patient cohorts and were shown to significantly predict outcomes after liver resection. Here, we show that these scores can also partly predict post-EH outcomes. However, the sensitivity and specificity of the different risk scores were heterogeneous, and none could satisfactorily predict mortality in patients who underwent EH. Therefore, we proposed a stepwise (two-stage) risk assessment strategy for predicting mortality in patients undergoing EH. Based on our sensitivity and specificity results, we suggest a two-stage Heidelberg−FIB-4 model, as shown in Fig. 4.

There are some limitations to the present study. This is a retrospective study, which should be validated by further prospective studies including only patients undergoing major hepatectomy. Furthermore, the Heidelberg score was developed in the same center. However, 3/4 of patients reported in the present study were used to develop the Heidelberg score. Also, the Heidelberg score was developed based on a cohort of 1,796 patients who underwent all types of liver resections (minor or major hepatectomy) and not just EH. The score was externally validated in a cohort of 281 patients from another center. Additionally, decisions about surgery based on the proposed scoring system should be made cautiously. Further trials are needed to evaluate the impact of one-stage surgery vs. two-stage surgery or associating liver partition and portal vein ligation for staged hepatectomy (ALPPS) in patients deemed “at-risk”. The present risk stratification can, however, help surgeons to at-risk patients before surgery. This will help prepare surgeons for providing special intra- and postoperative care, such as expert intraoperative anesthetic care, individual evaluation of the surgical approach (one- vs. two-stage hepatectomy, transection method, Pringle maneuver, etc.), and postoperative ICU care.

In conclusion, we have shown that no single risk assessment score can predict mortality in patients undergoing EH. Although mortality was predicted with acceptable discrimination, the sensitivity and specificity of the different tests were highly heterogeneous. Therefore, we have proposed a two-stage screening strategy using the risk scores with the highest sensitivity and specificity (Heidelberg−FIB-4 model) for patients undergoing major liver resection. Patient selection strategies are different in hepatobiliary centers across the world, so a multicenter prospective evaluation with higher sample sizes and a simultaneous assessment of predictive risk assessment scores is needed to determine which score can predict mortality following major liver resection.

## Methods

### Study design

Relevant data of all consecutive patients who underwent liver resection between January 2001 and January 2019 were investigated from a prospectively collected database. Only adult patients who underwent EH were included in this study. EH was defined as resection of five or more hepatic segments, based on the Brisbane 2000 classification^35^. A total of 276 patients were entered in the analysis. Twenty-six patients were excluded because data on the parameters used for all risk scores were missing. In the end, 250 patients were included in the final analyses. This study was approved by the independent ethics committee of the University of Heidelberg (approval number: S-754/2018). The requirement for informed consent was waived by the independent ethics committee of the University of Heidelberg because of the retrospective nature of the study. All procedures were conducted in accordance with the most recent revision of the Declaration of Helsinki.

### Study endpoints

The primary endpoint of this study was the ability of each test score to predict the risk of postoperative mortality after EH. To assess the discriminatory value of each test, the best cut-off point was evaluated, as well as the sensitivity and specificity of each test at predicting mortality after EH. The secondary endpoint was all-cause death occurring within 90 days after EH. The distribution of major morbidity in different risk groups (low-, intermediate-, and high-risk groups) was also assessed. Major morbidity was defined as any grade IIIb–IV complications (based on the Clavien–Dindo^36^ classification) that occurred within the first 90 days after surgery.

### Determination of well-known risk scores

To find relevant risk scores for predicting mortality after EH, high-impact hepatobiliary surgery or hepatogastroenterology journals were systematically searched. Journals were *Annals of Surgery, JAMA Surgery, British Journal of Surgery, Journal of the American College of Surgeons, Journal of Hepato-Biliary-Pancreatic Sciences, Annals of Surgical Oncology, Surgery, Journal of Hepatology, Hepatology, American Journal of Gastroenterology, Liver Cancer, Clinical Gastroenterology and Hepatology, The Lancet Gastroenterology and Hepatology, and Liver International*. Risk scoring systems that were reported in one of the above-mentioned journals and were defined or validated in more than 500 patients were identified. The parameters needed to assess each score were evaluated and risk assessment scores that included parameters that were not available in the institutional database were excluded. The included risk assessment scores were the aspartate aminotransferase-to-platelet ratio index (APRI)^25^, albumin to bilirubin (ALBI) grade^13,37^, predictive score developed by Breitenstein *et al*.^31^, liver fibrosis (FIB-4) index^12,30^, Heidelberg score^29^, and MELD score^33^. Risk assessment scores are presented in Table 3.

### Statistical analysis

Statistical analysis was performed using IBM SPSS Statistics for Windows, Version 24.0 (IBM Corp. Released 2013. Armonk, NY). Categorical data are presented as frequencies and proportions, and continuous data as means ± standard deviations. Categorical data were compared using chi-square test of association or Fisher’s exact test. Continuous data were compared using Student’s t-test. The proportion of morbidity and mortality in each risk assessment score was calculated and best curve estimations were made. The rate of major morbidity and 90-day mortality were compared between low-, intermediate-, and high-risk groups defined by each risk assessment score. ROC curve analysis and diagonal reference lines charting were used to compute the cut-off value that best discriminates the 90-day mortality risk between groups, as well as sensitivity and specificity of each risk assessment score for 90-day mortality. Cut-off points were identified by Youden’s J statistic. Pairwise comparison of AUC of risk assessment scores was performed using the method of DeLong *et al*.^38^ and MedCalc version 19.0.3 (MedCalc Software, Inc., Mariakerke, Belgium). A two-sided *p* value less than 0.05 was considered significant in all analyses.

## Data Availability

The datasets generated and/or analyzed during the current study are available from the corresponding author on reasonable request.
